# P-1381. Gender-based Testing Differences for *Chlamydia trachomatis* and *Neisseria gonorrhoeae* in Military Service Members

**DOI:** 10.1093/ofid/ofae631.1557

**Published:** 2025-01-29

**Authors:** David Manuel Aleman-Reyes, James Aden, Miguel A Arroyo Cazurro, Joseph Marcus

**Affiliations:** San Antonio Uniformed Services Health Education Consortium, San Antonio, Texas; BAMC, San Antonio, Texas; Brooke Army Medcial Center, Ft. Same Houston, Texas; Brooke Army Medical Center, San Antonio, TX

## Abstract

**Background:**

*Chlamydia trachomatis* and *Neisseria gonorrhoeae* infections impose a significant burden to the military, due to its young, sexually active population. In previous studies, female service members had 3-5 times the rate of both these infections as compared to men for unclear reasons. The purpose of this study was to investigate if gender-based differences in infection rates for chlamydia and gonorrhea may be due to gender-based differences in testing practices.

Demographic Information of 1620 Military Service Members Tested for Chlamydia trachomatis and Neisseria gonorrhoeae at Brooke Army Medical Center and Wilford Hall Ambulatory Surgical Center June-September 2023

**Figure:**
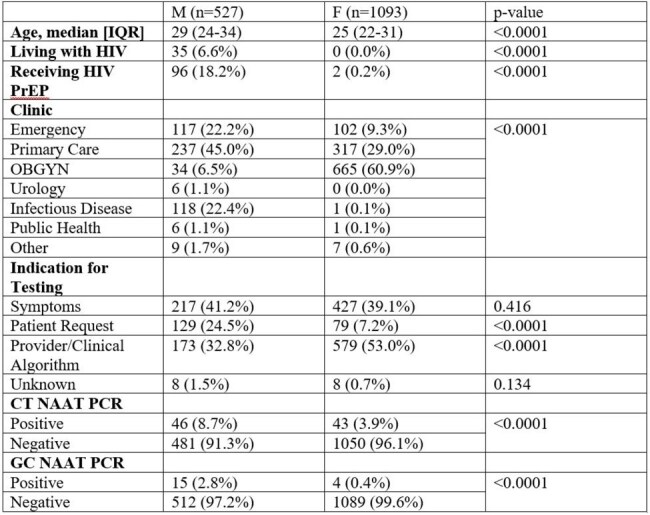

**Methods:**

A retrospective chart review was conducted on military service members who underwent testing for chlamydia and gonorrhea at Joint Base San Antonio between June and September 2023. The local electronic health record database was queried to determine patient demographics (age, gender, race, ethnicity, rank, military branch), clinical setting and indications for testing.

**Results:**

A total of 3768 patients were identified. 2148 female basic trainees who underwent mandatory entry screening were excluded, leaving 1620 (43%) patients for analysis. The cohort was predominantly female (67.5%) and enlisted (84.2%) with a median age of 27. Male patients were more likely to be tested for patient driven factors, such as symptoms (41.2%) or patient request (24.5%). Female patients were most frequently tested due to clinical algorithm (53.0%). Tested male patients were more likely to have a positive result than female patients for both chlamydia (8.7% vs 3.9%, p=< 0.001) and gonorrhea (2.8% vs 0.4%, p=< 0.001) (Table).

**Conclusion:**

Although women were more frequently tested for chlamydia and gonorrhea infections, male service members had significantly higher positivity rates, with more patient-driven indications for testing. This study implies the higher positivity rates in female patients compared to the male patients that has been previously reported is at least partially due to a testing bias.

**Disclosures:**

**All Authors**: No reported disclosures

